# Acid-Treated Water-Soluble Chitosan Suitable for Microneedle-Assisted Intracutaneous Drug Delivery

**DOI:** 10.3390/pharmaceutics11050209

**Published:** 2019-05-02

**Authors:** Ajeesh Chandrasekharan, Young Jun Hwang, Keum-Yong Seong, Samdae Park, Sodam Kim, Seung Yun Yang

**Affiliations:** 1 Department of Biomaterials Science, Life and Industry Convergence Institute, Pusan National University, Miryang 50463, Korea; ajeechem@gmail.com (A.C.); hwangyj@snvia.com (Y.J.H.); ky.seong0124@gmail.com (K.-Y.S.); damsoho@gmail.com (S.K.); 2 SNvia Co., Ltd, Busan 46241, Korea; park3dae@snvia.com

**Keywords:** chitosan, microneedle, drug delivery, transdermal patch

## Abstract

Chitosan has been widely used as a nature-derived polymeric biomaterial due to its high biocompatibility and abundance. However, poor solubility in aqueous solutions of neutral pH and multiple fabrication steps for the molding process limit its application to microneedle technology as a drug delivery carrier. Here, we present a facile method to prepare water-soluble chitosan and its application for sustained transdermal drug delivery. The water-soluble chitosan was prepared by acid hydrolysis using trifluoroacetic acid followed by dialysis in 0.1 M NaCl solutions. We successfully fabricated bullet-shaped microneedle (MN) arrays by the single molding process with neutral aqueous chitosan solutions (pH 6.0). The chitosan MN showed sufficient mechanical properties for skin insertion and, interestingly, exhibited slow dissolving behavior in wet conditions, possibly resulting from a physical crosslinking of chitosan chains. Chitosan MN patches loading rhodamine B, a model hydrophilic drug, showed prolonged release kinetics in the course of the dissolving process for more than 72 h and they were found to be biocompatible to use. Since the water-soluble chitosan can be used for MN fabrication in the mild conditions (neutral pH and 25 °C) required for the loading of bioactive agents such as proteins and achieve a prolonged release, this biocompatible chitosan MN would be suitable for sustained transdermal drug delivery of a diverse range of drugs.

## 1. Introduction

Transdermal drug delivery systems (TDDSs) have been gaining popularity as a potential alternative to oral administration and hypodermic injection because of their advantages such as better patience compliance, reduced toxicity, and increased dosage efficacy [1,2,3,4]. However, the greatest barrier to transdermal drug delivery is the low permeability of skin. In particular, the outermost layer of the skin (stratum corneum) acts as a significant permeation barrier. This layer consisting of corneocytes embedded in lipids regulates the permeability of drugs through skin, which is influenced by various physiochemical properties of the drug such as the octanol–water partition coefficient with appropriate lipophilicity, molecular weight (MW) < 500 Da, ionization, and solubility. In addition, physiological properties such as age, hydration, and temperature influence the rate of permeation through the skin [5,6,7,8]. To overcome these limitations, recent developments in TDDSs have focused on enhancing the permeability of skin to facilitate the transport of hydrophilic drugs and biomacromolecules including proteins and peptides [9,10,11]. To enhance the permeability of skin, various approaches using chemical enhancers, sonophoresis, iontophoresis, and nanocarriers have been developed [12,13,14,15,16]. However, the use of chemicals might cause skin irritation, while ultrasound or high voltage to increase drug permeation could result in damage to the deeper tissues. Transdermal drug delivery using nanocarriers is effective to deliver both hydrophilic and hydrophobic drugs, but toxicological studies are required to transfer this technology into clinical applications [17,18]. 

Recently, microneedle (MN)-assisted TDDSs have been widely investigated as they could create micron-sized holes, which provide a direct channel of mass transport regardless of drug type. Depending on the type of drug formulation and desired pharmacokinetic response in a body, numerous MN arrays had been fabricated in various sizes and geometries with diverse materials [19,20,21,22]. Although metal MNs prepared using silicon or ceramics can enhance the permeability of skin [23,24], complicate fabrication procedures employing conventional lithography, poor drug loading capacity, and health hazards arising from the breakage of these materials are limitations [25,26].

To overcome these issues, polymer-based MNs have been used, as they exhibit better biocompatibility and can be cost-effectively fabricated on a large scale. In addition, this system allows higher drug loading capacity as the drugs can be loaded on the tip, base or coated on the needle. A wide range of synthetic or natural polymers have been used to tailor the mechanical properties, drug release profile, and biocompatibility of the MNs [27,28,29,30,31]. Nevertheless, biodegradable synthetic polymers such as poly(lactic-*co*-glycolic acid) are soluble in organic solvents or require high temperatures to melt and process the materials [32,33,34]. These conditions could damage sensitive bioactive therapeutics such as proteins and peptides, whereas synthetic water-soluble polymers such as poly(vinylpyrrolidone) and poly(vinyl alcohol) have been used for MN fabrication under mild conditions, allowing a wide range of therapeutics to be encapsulated. The MNs prepared from these water-soluble polymers were effective for fast release of the encapsulated drug following insertion into skin [35]. Recently, for better biocompatibility, water-soluble natural polymers have gained special attention as MN materials [36]. In particular, clinically relevant (human injectable) polymers such as hyaluronic acid [37] are advantageous for relatively easy translation to clinical application when used in MN fabrication as a drug delivery vehicle. The MN-array patches offer a minimally invasive and less painful method of drug delivery, hence better patience compliance over the conventional parenteral injection method. In this regard, nature-derived polymeric biomaterials have been widely studied [38]. To control the drug release and mechanical properties, water-soluble polymers are physically or chemically crosslinked. While the crosslinking approach was effective to achieve a sustained drug release, several issues related to the toxicity arising from chemicals used for the crosslinking process and the possible damage to the fragile bioactive therapeutics encapsulated need to be resolved [39,40]. 

Chitosan (CS) is a natural linear polysaccharide derived from the partial deacetlylation of chitin. It is composed of randomly distributed β-(1-4)-linked *D*-glucosamine and *N*-acetyl-*D*-glucosamine units in the polymer [41]. Owing to its excellent biocompatibility and biodegradability, it has been used in various biomedical applications such as tissue engineering and wound dressing. Importantly, the use of CS in humans as a dietary supplement and drug carrier has been found to be safe [42,43]. However, CS exhibits poor solubility in neutral water owing to the presence of basic NH_2_ groups [44]. This group in the CS polymeric chain has a pK_a_ value of 6.3 to 6.6, depending on the polymer chain length and degree of deacetylation (DDA). Above the pK_a_ of CS, the NH_2_ groups remain unprotonated, leading to possible inter-/intra-molecular hydrogen bonds between polymeric chains and eventually precipitation [45]. Hence, slightly acidic conditions are required to dissolve CS polymers, especially for those with MW > 30 kDa [46]. Although CS can be dissolved in acetic acid solutions (1–3 *v*/*v*% in water), the presence of acid residues can lower the biocompatibility of the system and might affect the activity of encapsulated drugs [41].

To obtain a water-soluble chitosan (WSCS), several methods such as oxidative, enzymatic or acid hydrolysis have been used [44,47,48,49]. The oxidative hydrolysis of CS using hydrogen peroxide has been used to prepare WSCS. However, this approach could result in structural changes such as deamination and ring opening of the disaccharide unit leading to the formation of carboxyl groups, especially for low-MW CS [48], whereas the cleavage of polymer chains using enzymes was effective to increase the solubility of CS in neutral water (pH 7.0) without significantly affecting the CS structural unit. However, each enzyme requires an optimum temperature and pH range for a long time (up to 24 h) [50]. In addition, cost and limited availability of the CS-specific enzymes make this process commercially less attractive [47]. Alternatively, acid treatment of CS has been used to obtain WSCSs because it increases water solubility without causing significant change in the basic skeletal disaccharide unit and DDA [51]. Acid hydrolysis mainly involves the scission of *O*-glycosidic linkage, resulting in the cleavage of polymer chains to form smaller fragments, thereby minimizing the change in chemical nature related to the biological activities of CS [52]. Typically, WSCSs have been prepared by acid hydrolysis using hydrochloric acid, nitrous acid, or phosphoric acid [41,51]. However, the WSCS prepared by previous acid hydrolysis conditions is difficult to apply as a MN material due to reduced mechanical properties and difficulty to remove byproducts arising from browning reactions, which is generated during hydrolysis in strong acids. 

In this work, we report a facile method to increase the water solubility of CS in neutral pH by a simple treatment with trifluoroacetic acid (TFA), resulting in the random cleavage of glycosidic bonds in CS chains. After the complete removal of byproducts by dialysis in 0.1 M sodium chloride (NaCl) solutions, the TFA-treated WSCS was used to prepare a bullet-shaped MN array for transdermal drug delivery. It was found that the acid treatment was effective to prepare 7 wt % aqueous CS solutions with good filling properties, required for MN fabrication based on solvent casting. CS MN arrays have been prepared from WSCS solutions by the single molding process without significant shrinkage from the mold. The mechanical properties, skin penetration, and water responsiveness of the CS MN was investigated to evaluate its efficacy as a MN material. Furthermore, drug release kinetics of the CS MN using a model hydrophilic drug (rhodamine B) was examined using a static Franz diffusion cell. 

## 2. Materials and Methods 

### 2.1. Materials

CS (low-MW CS, DDA: >75 %), deuterium oxide, deuterium chloride, and acetic acid-d_4_ for nuclear magnetic resonance (NMR) measurements, and rhodamine B (MW: 479.01; λ_ex_: 553 nm; λ_em_: 627 nm; octanol–water partition coefficient (log K_OW_): 1.95) were purchased from Sigma Aldrich (Seoul, Korea). The following chemicals were reagent grade and were used as purchased from Sigma Aldrich: acetic acid, sodium hydroxide, sodium chloride, hydrochloric acid, and sodium acetate. TFA was obtained from Junsei Chemical Co., Ltd. (Tokyo, Japan). The cellulose membrane (MWCO 3500) for dialysis was purchased from Membrane Filtration Products, Inc. (Seguin, TX, USA). Qualitative filter paper (Ø = 70 mm; pore size = 0.45 μm) was purchased from Whatman (Maidstone, UK). A liquid prepolymer (Sylgard 184A) and a curing agent (Sylgard 184B) for the polydimethylsiloxane (PDMS) molding were purchased from Dow Corning (Midland, MI, USA). Agar powder for the in vitro test was purchased from Samchun Chemical (Pohang, Korea).

### 2.2. Preparation of the WSCS by TFA Treatment

CSs (2 *w*/*v*%) were dissolved in TFA and stirred at 37 °C with different reaction times (30 and 90 min). The mixture was allowed to cool down to 25 °C and then dialyzed against 0.1 M NaCl solution at 25 °C for 48 h, until a stable pH (~6.0) was obtained. This was followed by dialysis against plain deionized (DI) water for 24 h. The dialyzed solutions were filtered, and the filtrate was lyophilized using a freeze dryer (HyperCOOL HC4055, Labogene, Korea) for 48 h to obtain a white solid. The acid-treated CS solids were stored at −20 °C until before use. CSs obtained after treatment with TFA for 30 and 90 min will be referred as WSCS30 and WSCS90, respectively.

### 2.3. Characterization of the WSCS

Carbon NMR (^13^C NMR) spectra were obtained from CS, WSCS30, and WSCS90 in 2 *v*/*v*% of CD_3_COOD/D_2_O using a 600 MHz FT-NMR spectrometer (600 MHz Agilent Superconducting FT-NMR Spectrometer System, Agilent, Santa Clara, CA, USA). Proton NMR (^1^H NMR) spectra were obtained for CS, WSCS30, and WSCS90 in 2 *v*/*v*% of DCl/D_2_O at 70 °C using a 500 MHz FT-NMR spectrometer (500 MHz Agilent Superconducting FT-NMR Spectrometer System, Agilent, Santa Clara, CA, USA). The DDA of CSs was calculated from ^1^H NMR spectrum using the procedure described in the literature [53]. 

### 2.4. Intrinsic Viscosity and Viscosity-Average MW 

To determine the intrinsic viscosity and viscosity-average molecular weight (M_v_), CS, WSCS30, and WSCS90 solutions were prepared by dissolving the polymers (0.1, 0.15, 0.2, 0.25, 0.3, 0.35, and 0.4 g/mL) in a solution containing 5 mL of 0.25 M acetic acid and 0.25 M sodium acetate. The intrinsic viscosity ([η]) by average efflux time of each sample was measured at 25 °C using an Ubbelohde viscometer (size: 50; capillary Ø = 0.44 mm; CANNON Instrument Company, State College, PA, USA). 

The relative viscosity (η*_r_*), which is the ratio of solution viscosity to solvent viscosity, was calculated using Equation (1) as follows:

(1) $$\eta_{r}=\frac{t_{a}}{t_{0}}$$

where *t*_a_ is the efflux time of the solution and *t*_0_ is the efflux time of the solvent. The specific viscosity (η*_sp_*), which is calculated from the increment in viscosity due to the polymer, was calculated using Equation (2):

(2) $$\eta_{sp}=\frac{\left(t_{a}-t_{0}\right)}{t_{0}}=\eta_{r}-1$$

Intrinsic viscosity ([η]) is defined as reduced viscosity (η*_red_*) extrapolated to a CS concentration (C) of zero as follows:

(3) $$\left[\eta \right]=\eta_{red}={\left(\eta_{sp}/C\right)}_{c\to 0}={\left(\eta_{red}\right)}_{c\to 0}$$

where C is in g/mL. The viscosity-average molecular weight (*M_v_*) of the CS was calculated using the Mark–Houwink equation (Equation (4)) [54]. K (0.00016) and a (0.79) are constants for a given solute–solvent system and temperature [55].

(4) $$\left[\eta \right]=KM_{v}^{a}$$

### 2.5. Solubility Test

To check the dissolution properties of TFA-treated and non-treated CSs, 1 wt % of CS, WSCS30, and WSCS90 were stirred in DI water at different pHs (2.6, 6.0, and 7.4) for 24 h at 25 °C and then photographed. The pH levels of the aqueous solutions were adjusted by using 1 M hydrochloric acid and 1 M sodium hydroxide solution. 

### 2.6. Zeta Potential Test

The zeta potential (ZP) of the CS solutions were measured at different pHs (5.0, 6.0, 7.0, and 8.0) using the zeta-potential measurement system (Nano ZS-90, Malvern Instruments, Malvern, UK) [46].

### 2.7. Fabrication for the Array of WSCS MNs with Bullet-Shaped Geometry

To fabricate the WSCS MN arrays, negative PDMS molds were prepared by replicating from the positive bullet-shaped MNs milled by using CNC machine [56]. The WSCS MN array (100 MN/1 × 1 cm^2^) was prepared by solvent-casting of 7 *w*/*v*% WSC30 solution in DI water (pH 6.0) following a degassing step in a vacuum chamber at 25 °C to fill the cavities of the negative PDMS mold. The CS MN patch was dried at 25 °C for 24 h and gently peeled from the PDMS mold. The MN patches were visualized using digital microscopy (Dino-Lite, AM 413TL, Taiwan) and field-emission scanning electron microscopy (FE-SEM, Hitachi-4700, Tokyo, Japan). 

### 2.8. Mechanical Tests of the WSCS MNs

An axial fracture test using a Universal testing machine (AND, A&D 5000H, Daegu, Korea) was used to determine the mechanical properties of CS MNs. First, a single CS MN was horizontally attached to a pin stub using an adhesive. The metal pin stub was fixed horizontally in an opposing position, and then moved at a rate of 0.1 mm/min. The fracture force was measured by loading until failure. The force (N) and displacement (mm) data were measured to determine the fracture force of the CS MN. To determine if the MNs could pierce skin without fracturing, arrays of CS MN arrays were pressed into pig skin. After removing MNs, puncture marks on the skin were characterized using a digital microscope system.

### 2.9. In Vitro Dissolution Tests of the WSCS MNs

The agarose gels were prepared by dissolving agar powder in DI water to form 3 and 7 wt % solutions. The 3 wt % solution was poured into a 1-mm thick mold and the 7 wt % solution was poured into a 20-μm thick mold. The molded agarose gels were cooled to 25 °C and stacked so that the 7 wt % gel was above the 3 wt % gel; the molds were then adhered to each other with an instant adhesive. The dissolution of the WSCS MNs following insertion into the double-layered agarose gels was monitored using a TS100 Nikon Eclipse system (Nikon, Tokyo, Japan).

### 2.10. Ex Vivo Drug Release from the WSCS MN Patches

To investigate the ex vivo permeation kinetics across the skin of rhodamine B released from CS MNs, static diffusion experiments were conducted using a Franz diffusion cell. Pig skin (hair removed) was purchased from a local butcher shop and kept refrigerated at −20 °C until used in the experiment. Before performing the drug release tests, the pig skin was thawed at 25 °C for 1 h and prepared by carefully removing the subcutaneous fat using a single-edge blade (Dorco co., Seoul, Korea). To fabricate rhodamine-loaded CS MN patches, MN-forming solution was prepared by adding rhodamine B in 7 wt % WSCS30 aqueous solution to 0.02 wt %. The rhodamine-loaded CS MN patches were fabricated by the solvent-casting of a 7 wt % MN-forming solution (0.2 mL) with rhodamine on the PDMS mold with a 10 × 10 array of bullet-shaped cavities. After degassing and drying at 25 °C for 24 h, the rhodamine-loaded CS MN patch was peeled from the mold. The mass of MN tips in the patch (5 × 5 MN array) was measured after cutting them with a single-edge blade and the loaded amount of rhodamine in the MN tips was determined by measuring the fluorescence of rhodamine following dissolutions of the MN tips in DI water. The rhodamine-loaded CS MN patch (5 × 5 MN) was inserted on 3 × 3 cm excised ~1-mm thick pig skin. The rhodamine-loaded CS MN patch was inserted into the pig skin and a hydrocolloid adhesive patch (NeoDerm Roll, EVERAID, Yangsan, Korea) was applied on it during drug release. WSCS30 solutions of 50 μL with equal amounts of rhodamine B loaded in CS MNs was dropped on the pig skin as a control. The pig skin that applied the CS MN patch and rhodamine B solution was positioned between the donor and receptor chambers in the Franz diffusion cell. The donor chamber of the Franz cell was sealed with an aluminum foil to minimize solvent evaporation and photobleaching of dyes from the light. The receptor chamber with a side arm was filled with 20 mL of fresh PBS buffer (pH 7.4) and maintained in the water bath of 37 °C. After taking 1 mL in the receptor chamber at predetermined time points (0.5, 1, 2, 4, 6, 12, 24, 36, 48, 72, 96, and 108 h), 1 mL of fresh PBS buffer was refilled into the receptor chamber [23]. The samples obtained at each time point were dropped into the 96-well black polystyrene microplate and measured using a microplate reader (GloMax^®^, Promega, Medison, WI, USA) with a fluorescence detection module (λ_ex_: 520 nm and λ_em_: 580–640 nm). Standard rhodamine solutions in the range of 0.122–15.625 μg/mL were analyzed to quantify the amount of permeated rhodamine. 

## 3. Results and Discussion

### 3.1. Preparation and Characterization of the WSCS

**Figure 1:**
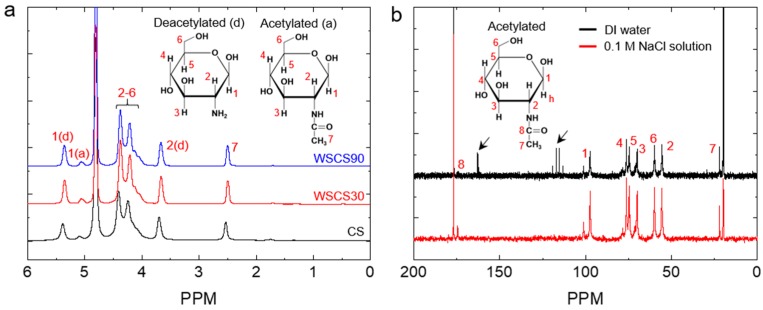
(**a**) ^1^H NMR spectra of CS, WSCS30, and WSCS90 in DCl/D_2_O. (**b**) A comparison of ^13^C NMR spectra of WSCS30 in CD_3_COOD/D_2_O obtained after dialysis against DI water (top) and 0.1 M NaCl solution (bottom), respectively. The peaks for trifluoroacetate ions are indicated by arrows. WSCS—water-soluble chitosan.

To prepare the WSCS by a simple acid treatment, we used a TFA for the hydrolysis of CSs without chemical modification of the basic disaccharide units in the polymeric CS chains. CSs were treated with TFA for 30 and 90 min at 37 °C to investigate the effect of reaction time on chemical structure and solubility in water. To remove trifluoroacetate ions, the reaction mixture was dialyzed against water and the pH was monitored until the solution reached a stable pH (~6.0). ^1^H NMR spectrum of WSCS30 and WSCS90 matched with that of the CS, suggesting that the disaccharide units remained unaffected (Figure 1a). However, the ^13^C NMR spectrum showed the presence of trifluoroacetate ion at 162.8 and 116.2 ppm [49], indicated by arrows in Figure 1b. The trifluoroacetate ion in CS solutions might cause safety issues when the polymer solution is used to fabricate medical devices including MN patches. To completely remove the trifluoroacetate ion, WSCS30 and WSCS90 solutions were thoroughly dialyzed against 0.1 M NaCl solution and subsequently in plain DI water, resulting in the formation of sodium trifluoroacetate, which can eventually be removed during dialysis against water [57]. As anticipated, trifluoroacetate ion peaks were not observed after dialysis in 0.1 M NaCl solution as shown in Figure 1b. The DDA of CS, WSCS30, and WSCS90 was determined from ^1^H NMR spectra (Table 1). The DDAs of TFA-treated samples were slightly higher (2.6%) than that of the CS while the deacetylation rate difference between WSCS30 (80%) and WSCS90 (81%) was not significant. This result indicates that the use of TFA resulted in a limited hydrolysis of the N-acetyl linkage and the rate decreased with TFA treatment time. Acid hydrolysis of CS involves the scission of both *O*-glyosidic linkage (main chain scission) and *N*-acetyl linkages (side chain scission) but, under strong acidic conditions, the cleavage of the glycosidic bonds is 10-fold higher than that of *N*-acetyl linkage [51]. From these results, we confirmed that the TFA treatment did not cause a significant change of chemical nature in CS.

Next, we investigated the MW change of CSs following TFA treatments by measuring the viscosity of CS solutions. The viscosity-average molecular weight (M_v_) of each sample was calculated from the intrinsic viscosity of samples by applying the Mark–Houwink equation (experimental Section 2.4). Unlike DDA trends, the intrinsic viscosity and M_v_ of the CS solutions considerably decreased with acid treatment time (Table 2), suggesting the rate of main chain scission was faster than that of the side chain scission. The TFA-treated CSs exhibiting low viscosity would be beneficial for MN fabrication, using solvent casting with a PDMS mold having micron-sized cavities.

**Figure 2:**
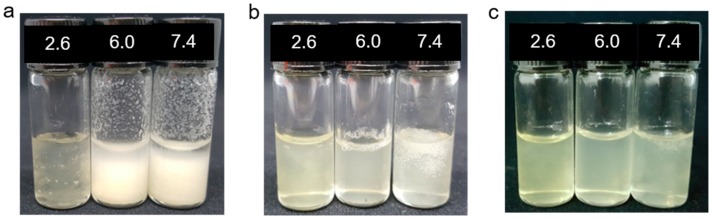
Solubility of (**a**) CS, (**b**) WCS30, and (**c**) WSCS90 at different pHs (2.6, 6.0, and 7.4).

The WSCSs obtained after acid hydrolysis need to have sufficient solubility in DI water for the single molding process. To check the solubility, 1 wt % of TFA-treated (WSCS30 and WSCS90) and non-treated CS samples were dissolved in aqueous solutions at different pHs (2.6, 6.0, 7.4) for 24 h, respectively. The non-treated CS was clearly dissolved in acidic solutions but exhibited poor solubility in solutions above pH 5.5 (Figure 2a). The TFA-treated CSs showed increased solubility at all tested pH ranges, particularly the WSCS90 which completely dissolved at pH 7.4 (above the pK_a_ of CS) as shown in Figure 2b,c. While the WSCS30 was not fully dissolved in water at pH 7.4, this polymer showed increased water affinity, thereby exhibiting water swelling behavior. 

The solubility of CS is determined by factors such as MW, DDA and the distribution of the acetyl group along the polymer chain of which MW plays a critical role. Table 3 shows the ZP of CSs at different pHs (5.0, 6.0, 7.0, and 8.0). The ZP of each CS decreased with increasing pH. In particular, a significant drop in pH values was observed at pH 7.0, as a fewer number of NH_2_ groups are protonated, whereas a higher ZP value was obtained at pH 5 and 6, as protonated groups (NH_3_^+^) are formed. A similar trend was previously reported by Chang et al., for CSs with MWs between 3 and 300 kDa [46]. Nevertheless, at a specific pHs, the CS exhibited lower ZP than WSCS30 and WSCS90. Considering the longer polymer chain length and lower DDA (78%) for CS, there is more possibility of a hydrogen bond and hydrophobic interactions between the polymeric chains, which could result in aggregation and subsequently precipitation. Tian et al., have reported that hydrogen bonds and hydrophobic interactions are the major reasons for the aggregation in CS, in which the former interaction is predominant [45]. With the decreasing protonation rate of NH_2_ groups in CS at pH 7, its solubility was decreased by increasing hydrogen bond interactions between the chains. Similarly, WSCS30 with a higher polymer chain length exhibited lesser solubility at neutral pH but showed higher ZP. This can be explained by the slightly longer polymer chain of WSCS30 and hence more NH_3_^+^ groups. Nevertheless, the DDA and M_v_ of CSs correlates well with the solubility experiment. For example, WSCS90 which has higher DDA and lower M_v_ exhibited better solubility at all tested pH values. In turbidity tests, TFA-treated CS solutions (WSCS30 and WSCS90) showed almost 10 times lower turbidity at pH 6, highlighting the increased solubility of CSs after TFA treatment (Figure S1 in Supplementary Information).

In the case of a polymeric MN system, the rate of drug release can be directly related to the strength of polymer matrix (gel strength) as this determines the rate of hydration and dissolution upon water (body fluid) uptake. The gel strength in turn is dependent on the MW of the polymer [58]. It was found that the higher the MW, the slower the rate of swelling and erosion, and subsequently slower drug release. For example, Learoyd et al., have shown that the higher MW (>310 kDa) CS powder exhibited a slower drug release compared to medium- (190–310 kDa) and low-MW (<190 kDa) polymers. They have also demonstrated how the rate of drug release can be tailored by choosing appropriate MWs of CS [59]. As controlled drug release over a longer period is desirable in MN-assisted TDDS, we selected the WSCS30 with increased water solubility at neutral pH and appropriate MW as a MN material.

### 3.2. Preparation of the CS MN Array Using WSCS30 Solutions

**Figure 3:**
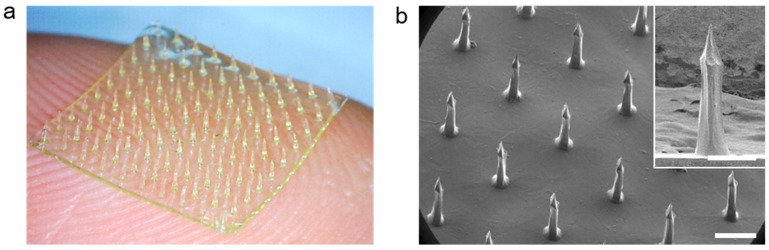
(**a**) A photograph and (**b**) tilted-view SEM images of the bullet-shaped CS MN patch (10 × 10 MNs/cm^2^), respectively. The CS MN array was reproducibly fabricated with dimensional uniformity (Scale bar; 500 μm).

The CS MN arrays were fabricated by using 7 *w*/*v*% of aqueous WSCS30 solution (pH 6.0) following solvent-casting on a PDMS mold with bullet-shaped cavities. The casting solutions showed good filling properties to the PDMS mold under vacuum and the polymer concentration was effective to form an MN array by the single molding process without significant shrinkage after drying (Figure 3a). The high pattern uniformity of the CS MN array with a density of 10 × 10 in 1 cm^2^ was examined by SEM measurement (Figure 3b). Each CS MN had a base diameter of 250 μm, a height of 750 μm, and a tip diameter of less than 10 μm. The bullet-shaped geometry of the CS MN was chosen as it provides better structural stability during skin penetration, optimal geometry for mechanical interlocking with tissue, and effective transdermal delivery of drugs encapsulated in MNs [38,56,60].

### 3.3. Fracture and Skin Insertion Tests Using the CS MNs

**Figure 4:**
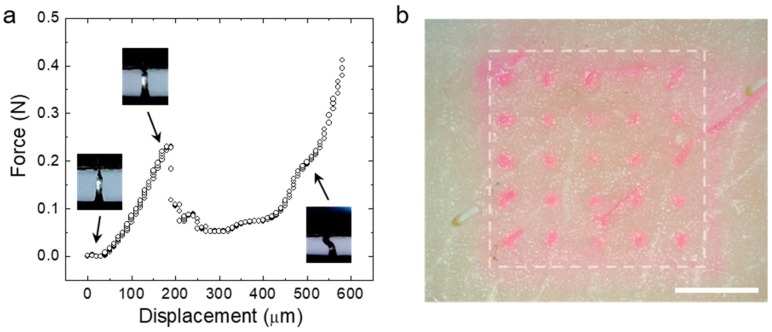
(**a**) The force displacement profile obtained from the axial compression test of the single CS MN. Snapshot images showing the response for the CS MN corresponding to the displacement point. (**b**) The photo image of the pig skin after the insertion test using the rhodamine dye-loaded CS MN array (Scale bar; 2 mm).

To provide enhanced transdermal drug delivery permeation, the CS MN array must have enough mechanical strength to penetrate the skin, thereby effectively delivering loaded drugs across the skin. To investigate mechanical properties of the CS MN, we conducted axial compression tests by pressing single MNs with a flat metal plate. Figure 4a shows the representative force-displacement curve obtained during compression of the MN. The force applied to the MN increased with displacement, then reached a maximum strength of ~0.25 N, preserving structural integrity of the MN. After this point, a compressive mode yield was measured without fracture. CS MNs prepared using less than 5 *w*/*v*% of aqueous WSCS30 solutions showed insufficient mechanical properties for tissue penetration. To confirm skin penetration of the CS MN, a MN array (5 × 5) loading rhodamine dye was applied to a pig skin with an insertion of 20 N for 1 min. After removing the dye-loaded MN array, we observed an array of dye spots corresponding to each needle, indicating insertion of the CS MNs into the skin (Figure 4b).

### 3.4. Water-Responsiveness of the CS MNs

**Figure 5:**
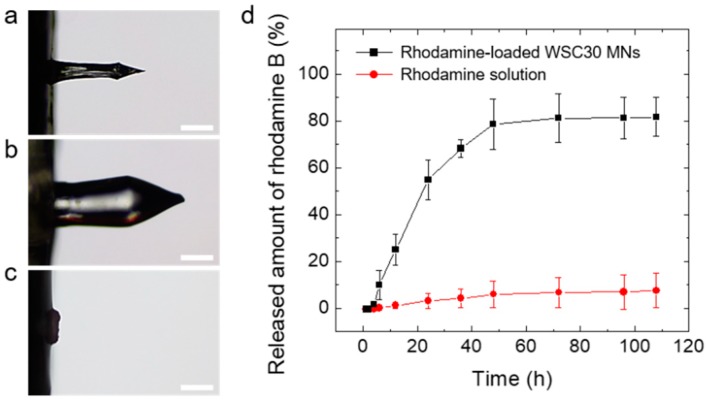
Optical microscope images of the CS MN (**a**) before swelling (dry state), (**b**) after insertion into the hydrogel for 2 min (maximum swollen state), and (**c**) complete dissolution in the hydrogel for 48 h (Scale bar; 250 μm). (**d**) In vitro permeation profiles of rhodamine B across skin obtained from CS MN patches (5 × 5 MN array) and topically applied rhodamine solutions, respectively. The permeation tests were performed using Franz diffusion cells (*n* = 3).

To investigate water-responsiveness of the CS MN, we monitored the dissolution behavior of the MN after insertion into a transparent double-layered hydrogel, mimicking different stiffnesses of the skin tissue. Interestingly, the CS MN did not immediately dissolve in the hydrogels but swelled due to water uptake, exhibiting a volume expansion of more than 5 times than that of its initial dry state (Figure 5a,b). After reaching its maximum swollen state in 2 min, the swollen CS MN was slowly dissolved in the course of 48 h (Figure 5c). This retarded dissolution behavior was likely attributed by physical crosslinking of CS polymeric chains [61,62]. Previously, several hydrogel-forming MNs have been prepared by using chemical crosslinking or physical crosslinking methods [39,40,63,64]. Although chemical crosslinking involving covalent bonds between polymer chains could provide a long-term structural stability against environmental variables such as pH, temperature, or ion strength, physically crosslinked hydrogels, especially prepared by water-soluble natural polymers, would be more biocompatible due to the lack of chemical crosslinkers [62]. The modulus of the swollen CS MN approximates that of skin tissue, which reduces the risk of underlying tissue damage with achieving the mechanical biocompatibility.

### 3.5. In Vitro Drug Release Tests

To determine the release kinetics of drugs loaded in the CS MN, rhodamine B (a hydrophilic model drug, red fluorescent dye)-loaded CS MN patches were inserted into pig skin and then the drug permeation across skin was monitored using a static Franz diffusion cell. Figure 5d shows the cumulative rhodamine permeation profiles across skin obtained from rhodamine-loaded CS MNs applied on the skin and topically administrated rhodamine solutions, respectively. The loading capacity of the rhodamine-loaded CS MN patch, defined as the mass of the loaded drug (rhodamine) in MN tips (2.2 ± 0.1 μg) divided by the mass of CS MN tips (341.7 ± 28.9 μg), was estimated to 0.6 ± 0.1 %. However, the loading capacity in the MN patch can be increased depending on drug concentration. As the drug in the MN-forming solution can be fully loaded in the MN patch without loss, the encapsulation efficiency, defined as the loaded drug amount in the tips (2.2 ± 0.1 μg) over drug quantity in MN-forming solutions (10 μg), was 22.1 ± 2.3 %. The rhodamine-loaded CS MN patches showed relatively fast linear release kinetics for the initial 24 h followed by a slow release rate over time. The permeated amount across skin was approximately 80 % of the total amount of loaded drugs (Figure 5d). In contrast, the rhodamine solution spread on the skin exhibited poor drug permeation, less than 10 % (0.85 ± 0.76 μg) of the total drug amount in the solutions. The drug release from a crosslinked hydrophilic matrix undergoes a complex process involving hydration and swelling of the matrix, followed by diffusion of the drug from it. The release kinetics of drugs loaded in the matrix can be facilitated by erosion of the matrix [58]. Considering the water-responsiveness and release kinetics of the CS MNs, drug release was dominantly governed by a swelling-controlled release mechanism for the initial period (24 h) followed by diffusion and an erosion-controlled release mechanism, which is typically observed in hydrogel-based drug delivery systems [58,62]. The drug could be loaded in the hydrogel-forming MN patch including the MN tips and the backing layer. The backing layer of the patch enables continuous drug delivery as a reservoir of the drug [65]. Therefore, not only the rhodamine loaded in the CS MN tips was released into the pig skin but also the rhodamine incorporated in the backing layer as a drug reservoir was delivered through the swollen CS MN tips. Hence, following insertion into the pig skin, the hydration of the CS MN matrix resulted in swelling and subsequently faster release as the drugs loaded at or near the surface were released. After reaching swelling equilibrium, the loaded drug would be released slowly as it diffused through the swollen matrix. This swellable CS MN would be beneficial in achieving prolonged transdermal delivery of drugs encapsulated in the MNs as the drug release occurs through the diffusion channels of swollen MNs following insertion into skin.

## 4. Conclusions

We have demonstrated a facile method to fabricate bullet-shaped CS MN arrays by the single molding process with neutral aqueous CS solutions and its application for sustained transdermal drug delivery. The WSCS was prepared by simple acid hydrolysis using TFA followed by dialysis with 0.1 M NaCl solution. The CS MN exhibited a sufficient mechanical strength for skin insertion and retarded dissolution behavior, thereby enabling sustained transdermal drug delivery. The rhodamine B, used as a model hydrophilic drug, loaded in the CS MN patch showed prolonged release kinetics in the course of the dissolving process for more than 72 h. Since the WSCS prepared by simple acid treatment can be used for MN fabrication in the mild conditions (neutral pH and 25 °C) required for the loading of bioactive agents such as proteins, this biocompatible CS MN would be suitable for sustained transdermal drug delivery of a diverse range of drugs.

## Figures and Tables

**Table 1:** DDA of CS, WSCS30, and WSCS90.

Samples	DDA (%)
CS	78
WSCS30	80
WSCS90	81

**Table 2:** Intrinsic viscosity and viscosity-average molecular weight (M_v_) of CS, WSCS30, and WSCS90.

Samples	Intrinsic Viscosity (dL/g)	M_v_ (kDa)^a^
CS	5.21	51.52
WSCS30	1.92	14.51
WSCS90	0.96	6.05

^a^ characterized by using [Equation (4)].

**Table 3:** Zeta potential of CS, WSCS30, and WSCS90 at different pHs.

Samples	Zeta Potential (mV)			
	pH 5.0	pH 6.0	pH 7.0	pH 8.0
CS	22.70 ± 1.13	14.33 ± 0.65	4.52 ± 0.15	−1.12 ± 0.27
WSCS30	29.95 ± 0.49	20.8 ± 0.61	6.89 ± 0.04	−0.21 ± 0.01
WSCS90	27.30 ± 1.87	16.43 ± 0.55	5.36 ± 0.34	−0.24 ± 0.07

## Supplementary Materials

The following are available online at https://www.mdpi.com/1999-4923/11/5/209/s1, Figure S1: The turbidity of CS, WSCS30, and WSCS90 solutions at pH 6.
