# Supplementary material for: Acid-Treated Water-Soluble Chitosan Suitable for Microneedle-Assisted Intracutaneous Drug Delivery
Source: Pharmaceutics. 2019 May 2;11(5):209. doi: 10.3390/pharmaceutics11050209 (PMC6572209; doi:10.3390/pharmaceutics11050209)
Supplement: Supplementary file 1 [file pharmaceutics-11-00209-s001.pdf]

pharmaceutics

Supplementary Materials

Acid-Treated Water-Soluble Chitosan Suitable for Microneedle-Assisted Intracutaneous Drug Delivery

Ajeesh Chandrasekharan, Young Jun Hwang, Keum-Yong Seong, Samdae Park, Sodam Kim and Seung Yun Yang

Turbidity Measurements

The turbidity of CS, WSCS30 and WSCS90 formed at pH6 was determined by measuring the solution turbidity with a 2100P portable turbidimeter (Hach, USA) as described in the literature [1]. 0.1w/v % of CS, WSCS30 and WSCS90 were dissolved in DI water and the pH was adjusted using 0.1 M hydrochloric acid and sodium hydroxide. The solutions were well homogenized by stirring for 10 minutes then kept idle for 30 min before measurements.

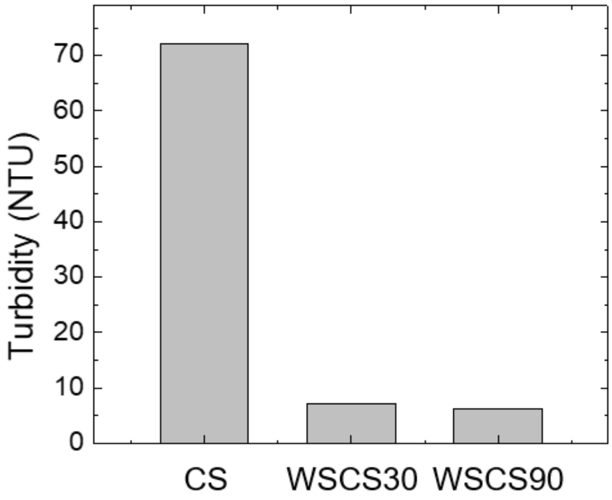

Figure S1. The turbidity of CS, WSCS30, and WSCS90 solutions at pH 6.

Refernce

1. Saïed, N.; Aïder, M. Zeta potential and turbidimetry analyzes for the evaluation of chitosan/ phytic acid complex formation. *J. Food Res.* **2014**, *3*, 71.
